# Myofibroblastic tumor (GIMT) in children: A case report

**DOI:** 10.1002/jpr3.70012

**Published:** 2025-03-31

**Authors:** Rui Wen, Jing Zhang, Pan Wang

**Affiliations:** ^1^ Department of Nuclear Medicine Affiliated Hospital of Zunyi Medical University Zunyi China

**Keywords:** differential diagnosis, endoscopy, macroscopic morphology

A 14‐year‐old girl, without any other clinical symptoms other than upper abdominal discomfort for 3 months, underwent upper gastrointestinal endoscopy. A large irregular mass with central ulceration (Figure 1A,B) was seen. Pathological examination and immunohistochemical examination demonstrated a gastric spindle cell tumor, and the final diagnosis was gastric inflammatory myofibroblastic tumor (GIMT, Figure 2A,B).

**Figure 1:**
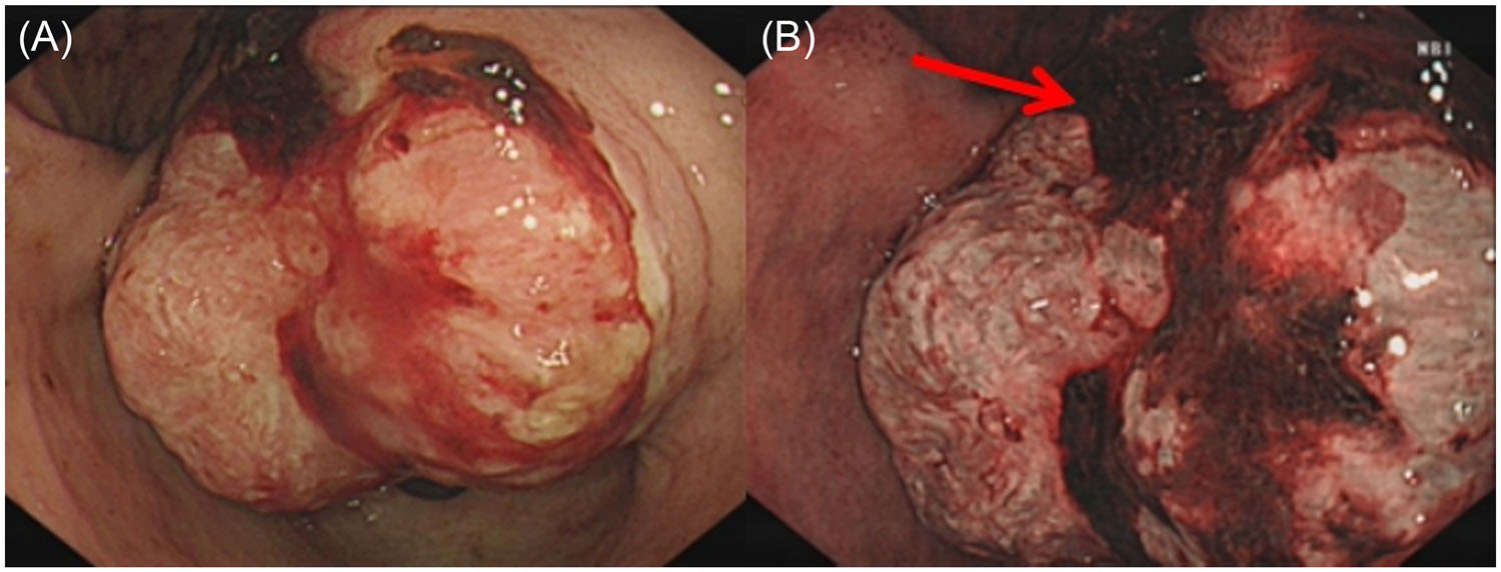
A large bleeding irregularly shaped submucosal mass (approximately 3 × 3 cm) with central ulceration was seen erupting through the gastric antral mucosa on the lessor curvature of the stomach. The surrounding antral mucosa was hyperemic and edematous (A, B, shown by the arrow).

**Figure 2:**
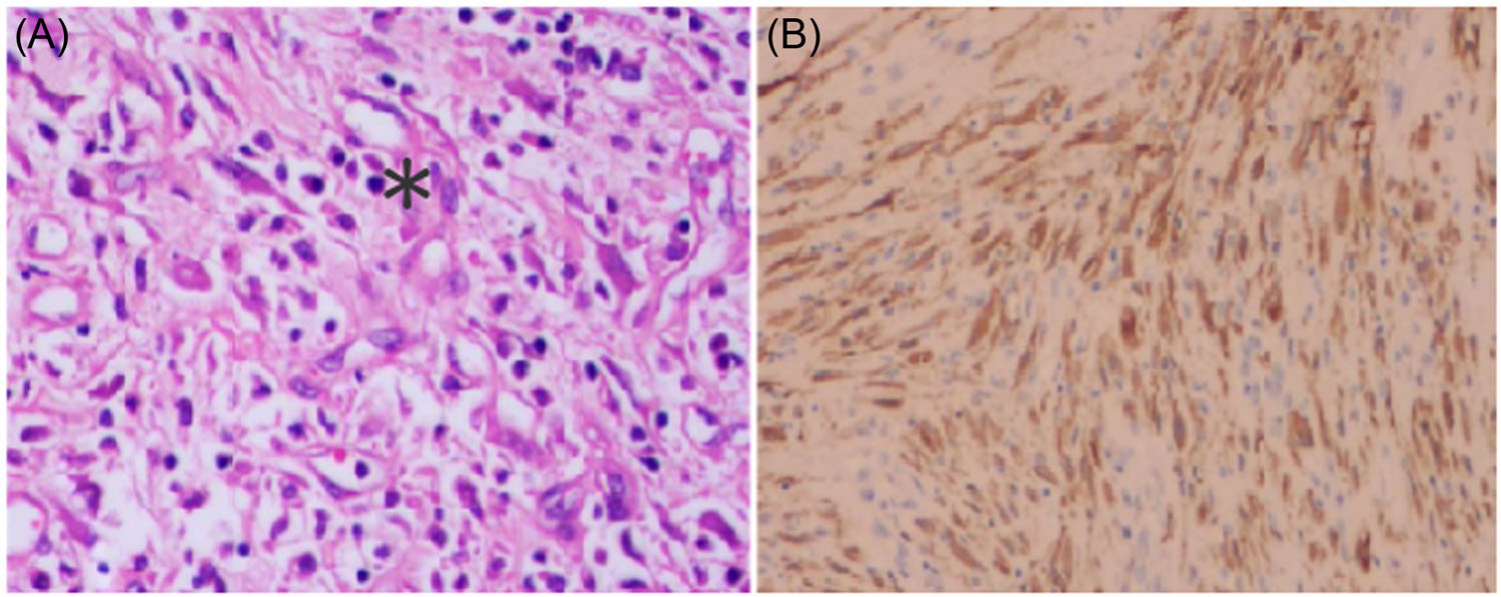
Histopathology: Gastric spindle cell tumor (*) was found in the lesser curvature of the stomach, and there was no tumor involvement in the distal and proximal resection margins of the stomach (A). Immunohistochemistry: tumor cell expression: ALK (+++), CD117 (−), SMA (+), Calponin foci (+++), SDHB (+), Ki‐67 (20%, +). Combined with morphology and immunohistochemistry, the final diagnosis of gastric inflammatory myofibroblastic tumor was established (classic, intermediate tumor).

GIMT is a rare and unique mesenchymal tumor, which often has low malignant manifestations or characteristics of borderline tumors. While it can occur at any age, there is a pediatric preponderance; it presents most commonly between 2 and 16 years old.^1^, ^2^, ^3^ It is rarely found in the esophagus or stomach. On endoscopic examination tumors typically appear as polypoid or elevated lesions that are mostly nodular or lobulated, with our without surface ulceration. Advanced tumors may demonstrate gastric wall invasion.^4^, ^5^ Histologically, GIMT are characterized by fibroblasts and spindle cell fibroblasts, and often accompanied with inflammatory infiltrates of a large number of plasma cells, lymphocytes, and eosinophil.^3^ The tumor cells are negative for CD117 expression and positive for SMA and ALK expression, whereas gastrointestinal stromal tumor tumors show the opposite profile.^6^ GIMTs have a relatively good prognosis but the literature reports a 1‐year recurrence rate of about 25%, and a distant metastasis rate of <5%.^7^ Complete surgical resection is the preferred treatment and chemotherapy may be required. Ongoing clinical follow‐up is needed, with regular imaging studies to check for recurrence.^8^

In this case, the patient's symptoms improved after laparoscopic subtotal gastrectomy and no recurrence was detected up to 1.5 years after surgery.

## CONFLICT OF INTEREST STATEMENT

The authors declare no conflicts of interest.
